# Occurrence of *Blastocystis* sp. in water catchments at Malay villages and Aboriginal settlement during wet and dry seasons in Peninsular Malaysia

**DOI:** 10.7717/peerj.2541

**Published:** 2016-10-06

**Authors:** Samseh Abdullah Noradilah, Ii Li Lee, Tengku Shahrul Anuar, Fatmah Md Salleh, Siti Nor Azreen Abdul Manap, Noor Shazleen Husnie Mohd Mohtar, Syed Muhamad Azrul, Wan Omar Abdullah, Norhayati Moktar

**Affiliations:** 1 Department of Parasitology and Medical Entomology, Universiti Kebangsaan Malaysia Medical Centre, Cheras, Kuala Lumpur, Malaysia; 2 Faculty of Medicine and Health Sciences, Universiti Sains Islam Malaysia, Pandan Indah, Kuala Lumpur, Malaysia; 3 Kulliyyah of Medicine and Health Sciences, Kolej Universiti Insaniah, Kuala Ketil, Kedah, Malaysia; 4 Department of Medical Laboratory Technology, Universiti Teknologi MARA, Puncak Alam, Selangor, Malaysia; 5 Integrative Pharmacogenomics Institute, Universiti Teknologi MARA, Puncak Alam Campus, Bandar Puncak Alam, Selangor, Malaysia; 6 Multipurpose Laboratory, Universiti Kebangsaan Malaysia Medical Centre, Cheras, Kuala Lumpur, Malaysia

**Keywords:** *Blastocystis* sp., Water, Aboriginal settlements, Wet and dry seasons

## Abstract

In the tropics, there are too few studies on isolation of *Blastocystis* sp. subtypes from water sources; in addition, there is also an absence of reported studies on the occurrence of *Blastocystis* sp. subtypes in water during different seasons. Therefore, this study was aimed to determine the occurrence of *Blastocystis* sp. subtypes in river water and other water sources that drained aboriginal vicinity of highly endemic intestinal parasitic infections during wet and dry seasons. Water samples were collected from six sampling points of Sungai Krau (K1–K6) and a point at Sungai Lompat (K7) and other water sources around the aboriginal villages. The water samples were collected during both seasons, wet and dry seasons. Filtration of the water samples were carried out using a flatbed membrane filtration system. The extracted DNA from concentrated water sediment was subjected to single round polymerase chain reaction and positive PCR products were subjected to sequencing. All samples were also subjected to filtration and cultured on membrane lactose glucuronide agar for the detection of faecal coliforms. During wet season, *Blastocystis* sp. ST1, ST2 and ST3 were detected in river water samples. *Blastocystis* sp. ST3 occurrence was sustained in the river water samples during dry season. However *Blastocystis* sp. ST1 and ST2 were absent during dry season. Water samples collected from various water sources showed contaminations of *Blastocystis* sp. ST1, ST2, ST3 and ST4, during wet season and *Blastocystis* sp. ST1, ST3, ST8 and ST10 during dry season. Water collected from all river sampling points during both seasons showed growth of *Escherichia coli and Enterobacter aerogenes*, indicating faecal contamination. In this study, *Blastocystis* sp. ST3 is suggested as the most robust and resistant subtype able to survive in any adverse environmental condition. Restriction and control of human and animal faecal contaminations to the river and other water sources shall prevent the transmission of *Blastocystis* sp. to humans and animals in this aboriginal community.

## Introduction

*Blastocystis* sp., a single-celled anaerobic enteroparasite inhabiting the lower gastrointestinal tract of humans and animals, has been reported to cause non-specific gastrointestinal symptoms (Souppart et al., 2010). The size of *Blastocystis* sp. is within the range of two known waterborne parasites *viz*, *Giardia* and *Cryptosporidium* (Suresh, Smith & Tan, 2005). *Blastocystis* sp. has been associated with two out of the 325 outbreaks (0.6%) of waterborne diseases caused by parasites worldwide (Karanis, Kourenti & Smith, 2007). In addition, *Blastocystis* sp. has been listed in the Water Sanitation and Health programmes of the World Health Organization and WHO Guidelines for Drinking-water Quality (World Health Organization, 2008; World Health Organization: microbial fact sheets, 2011).

Two studies in Malaysia have reported the occurrence of *Blastocystis* sp. in many water catchments, including recreational waters, rivers and lakes (Suresh, Tan & Illi, 2009; Ithoi et al., 2011). Absence of proper piped water supply was found to be a significant risk factor in the acquisition of *Blastocystis* sp. infection (Abdulsalam et al., 2012; Anuar et al., 2013). Drinking unboiled and untreated water have also been reported to be associated with *Blastocystis* infection (Leelayoova et al., 2004; Li et al., 2007).

Reports are still lacking on the occurrence of *Blastocystis* sp. subtypes from surface water and other water sources in various communities in Malaysia. The objective of this study was to ascertain the potential of water as source of acquiring *Blastocystis* sp. infections in these communities. Furthermore, the absence of studies on the seasonal influence on the occurrence of *Blastocystis* sp. subtypes in river water and other water sources has also steered the conduct of this research. It is hoped that this present study will add essential information on the occurrence of *Blastocystis* sp. subtypes in water and create awareness towards the role of surface water and other water sources in the dynamic transmission of *Blastocystis* sp. infections.

## Materials and Methods

### Study and sampling areas

Water samples were collected from a river, Sungai Krau from October 2014–November 2014 during wet season and June 2015 during dry season. Being located at the north-eastern part of Malaysia, the study areas were heavily flooded during wet season, where most of the houses located near the river were affected. The collection of the water samples in wet season was carried out 1 to 2 months before the heavy flood while the collection of water samples during dry season was carried out five months after the flood. February was documented as the month of the least amount of rainfall in Temerloh area in 2014 and 2015 as recorded by Malaysian Meterological Department. The north-eastern states of Peninsular Malaysia usually receives less rainfall from June to July (Malaysian Meterological Department (MetMalaysia), Ministry of Science, Technology Innovation (2015)). However, in certain regions of Malaysia, rainfall show different patterns because of several other factors, including geographical location. Although the study area is located in the north-eastern state of Peninsular Malaysia, based on the rainfall data, the study area receives minimum rainfall from June to July (first minimum rainfall) and in February (second minimum rainfall).

Due to heavy flooding which lasted until early of January 2015, collection of water samples within the second minimum rainfall period in February was not performed, since most of the villages in the study area were still affected by the flood and clean-up as well as rebuilding after massive floods took months to be accomplished.

Sungai Krau flows along the Malay and five aboriginal villages in Kuala Krau, Temerloh Pahang. Of all the aboriginal villages, the most upstream were Kampung Terbol, followed by Kampung Pian, Kampung Lubok Wong, Kampung Pasu and the most downstream Kampung Penderas. Most of the aborigines settled in Kampung Penderas with the widest land area. With the smallest land area, the least occupied village is Kampung Terbol.

**Figure 1:**
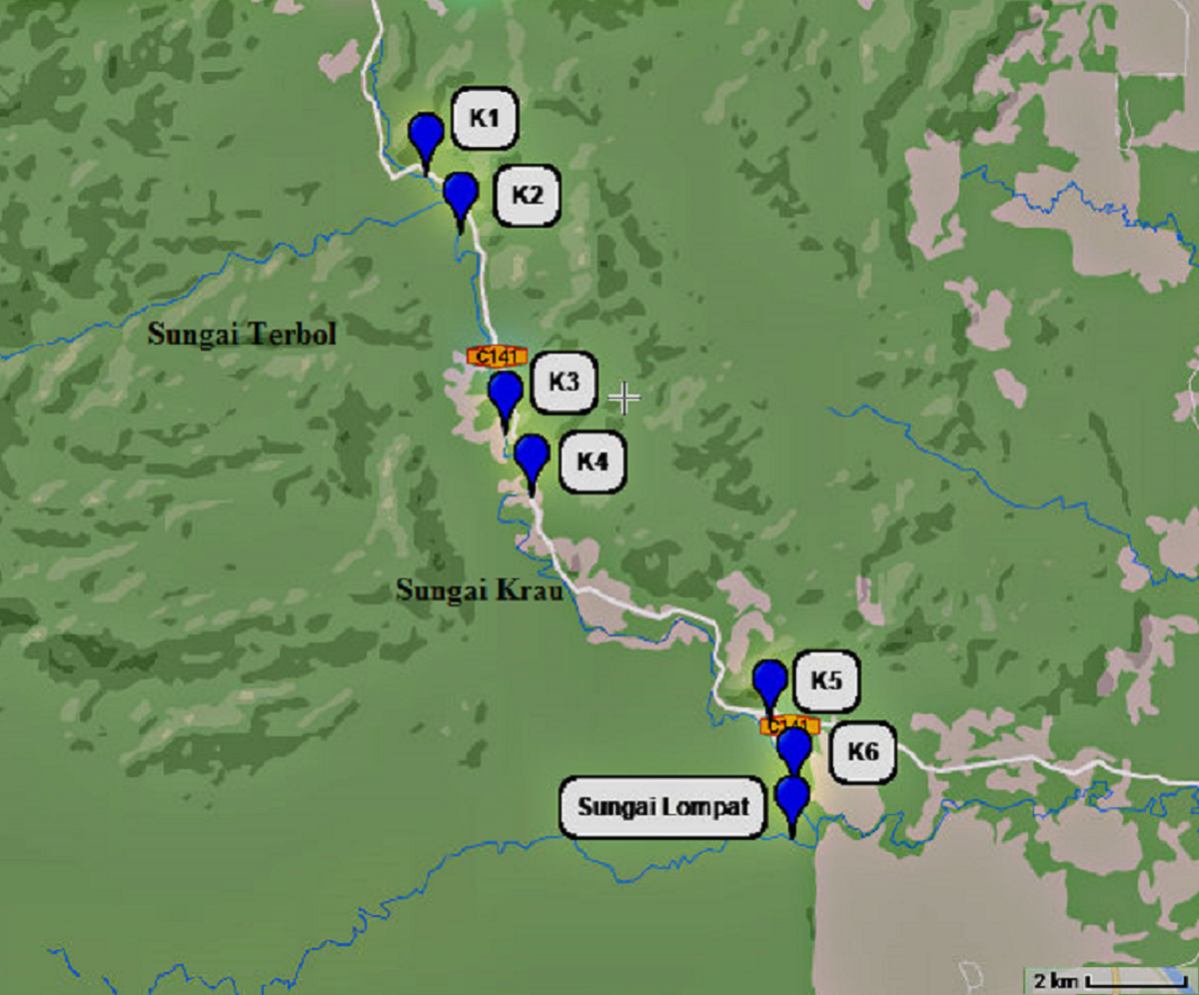
Map showing each six river sampling point at Sungai Krau and a point at Sungai Lompat.

Six sampling points of the river were identified; K1 (1,000 m before Kampung Terbol, 3.83507°, 102.21404°), K2 (in the middle of Kampung Terbol, 3.81314°, 102.22804°), K3 (1,000 m before Kampung Lubok Wong, 3.78516°, 102.23596°), K4 (in the middle of Kampung Lubok Wong, 3.77014°, 102.23763°), K5 (1,000 m before Kampung Penderas, 3.74364°, 102.27091°) and K6 (in the middle of Kampung Penderas, 3.71301°, 102.28753°). Another sampling point was determined at Sungai Lompat (3.71259, 102.28839), a river which flow and meets Sungai Krau downstream in Kampung Penderas (Figure 1). Other water sources were also determined, inclusive of wells, water tank, tap water and others.

### Collections of water samples

The study protocol has been approved by the Research and Ethical Committee, Faculty of Medicine, Universiti Kebangsaan Malaysia Medical Centre (FF-2014-219). Permission to conduct the sampling was obtained from the Ministry of Rural and Regional Development Malaysia, reference number : JAKOA/PP.30.032Jld29(04). Using 10 litres water containers, water samples were collected from each seven sampling points of the rivers. The sampling were performed about 10–15 feet from the river bank. The river water was collected from the surface of the river with extra caution to avoid floating material at the water surface.

Besides the collection of 10 litres of river water samples, one thousand and five hundred millilitres of river water were also collected at the same points of all river water sampling for faecal coliforms count.

One thousand and five hundred millilitres bottles were used to collect water from various other water sources that are available in the villages. In Kampung Terbol, other sources was sampled from a water tank provided by the Malaysian government, tap water and well.

Tap water, stored water in a container and water in a fish pond were sampled in Kampung Lubok Wong. In Kampung Penderas water samples were collected from tap water, stored water, wells and small stream. All the water samples were brought back to the Community Laboratory in the Department of Parasitology and Medical Entomology, Faculty of Medicine, Universiti Kebangsaan Malaysia Medical Centre with no addition of preservatives for processing.

### Physicochemical parameter of the rivers

Data on pH, conductivity, temperature, dissolved oxygen, and total dissolved solid were recorded at each sampling point at Sungai Krau (K1, K2, K3, K4, K5, K6) and a single point at Sungai Lompat using multiparameter (Hanna, USA, model HI 9829). Measurement of turbidity was performed using microprocessor turbidity meter (model HI 93703; Hanna Instruments, Woonsocket, RI, USA). Using a colorimeter (Thermo Scientific, Singapore, model Orion AQ4000), chemical oxygen demand (COD) and sulfate concentrations were measured. Measurement of total chlorine was carried out using a multiparameter bench photometer (model HI 83200; Hanna Instruments, Woonsocket, RI, USA). The results of all physicochemical parameters were recorded for correlation analysis with the presence of *Blastocystis* sp. subtypes in water samples collected from the rivers.

### Rainfall data

The wet and dry seasons were determined based on the monthly total rainfall data of 2010–2013 recorded from the nearest station to the study area (Temerloh station) and were obtained from the Malaysian Meteorological Department (MetMalaysia). The monthly total rainfall volume recorded during samples collection in the wet season were 116.8 mm–276.4 mm. Meanwhile, the total rainfall volume during samples collection in the dry season was 87.8 mm.

### Detection of *Blastocystis* sp. subtypes

#### Filtration of water samples

Ten liters of water sample from each sampling point and 1.5 L of water sample collected from each of the water sources available in the villages were filtered using flatbed membrane filtration system (Masterflex I/P, model XX80EL230; Millipore, Billerica, MA, USA) through mixed cellulose esters (MCE) membrane filter with a 1.2 µm pore size and 14 mm diameter. Using a cell scrapper, the water concentrate on the membrane filter was removed thoroughly and rinsed three times with phosphate buffered saline (PBS). The washings were then centrifuged at 1, 400 × *g* at room temperature for 10 min to obtain water concentrate. The supernatant was discarded until 5mL was left and kept in the cold room (Lee et al., 2012).

#### DNA extraction and amplification of the DNA using single round polymerase chain reaction (PCR) and sequencing

Extraction of the DNA from all water samples were performed using QIAamp^®^ Fast DNA Stool Mini Kit (Qiagen, Hilden, Germany) and followed the manufacturer’s instructions.

Amplification of the extracted DNA of all water samples were performed using BhRDr (genus-specific): GAGCTTTTTAACTGCAACAACG and RD5 (broad eukaryotic-specific): ATCTGGTTGATCCTGCCAGT primers (Scicluna, Tawari & Clark, 2006).

PCR was performed by 30 cycles of initial denaturing at 95 °C for 5 min, followed by denaturation at 95 °C for 1 min, annealing at 63.3 °C for 1 min and 30 s, extending at 72 °C for 1 min and an additional cycle of 10 min chain elongation at 72 °C. The PCR products were visualized in 1.5% agarose gel. PCR were carried out in duplicate to detect a possibility of mixed *Blastocystis* sp. subtype infections. Positive PCR products were then sent to Genomics Bioscience Taiwan for sequencing using the amplification primers to determine the subtypes. The sequences were then compared with the sequences available in GenBank™ using the BLASTN program on the National Center for Biotechnology Information Server (http://www.ncbi.nlm.nih.gov/BLAST). The sequences obtained from the sequencing were also deposited in GenBank™ (accession numbers: KX351998 –KX352032). The sequences were also queried into the “*Blastocystis* ST (18S) and Multi Locus Sequence Typing (MLST) Multi Locus Sequence Typing Databases” (www.pubmlst.org/blastocystis) which identified the sequences to 18S allele level (Stensvold, 2013). The exact or closest match allele to each sequence was identified.

### Detection of faecal coliforms

Approximately 100 µL, 10 µL, 1 µL and 0.1 µL of water samples from the rivers in 100 mL of phosphate buffered saline (PBS) were filtered through 0.45 µm, 5 cm diameter nitrocellulose membrane filter. The filter was transferred onto membrane lactose glucuronide agar (MLGA) and incubated at 37 °C for 24–36 h. Plates were observed daily until 36 h for the presence of faecal coliforms indicated by colours ; Green (*Escherichia coli*) and yellow (*Enterobacter aerogenes*) colonies. The colonies of both faecal coliforms were counted and the results were expressed in number of colonies in every 100 ml water. Both bacteria were faecal coliforms.

### Statistical analysis

A correlation analysis using Spearman’s rho was performed using a statistical software package (SPSS version 22) to determine the correlation between the physicochemical parameters and the occurrence of *Blastocystis* sp. subtypes.

**Figure 2:**
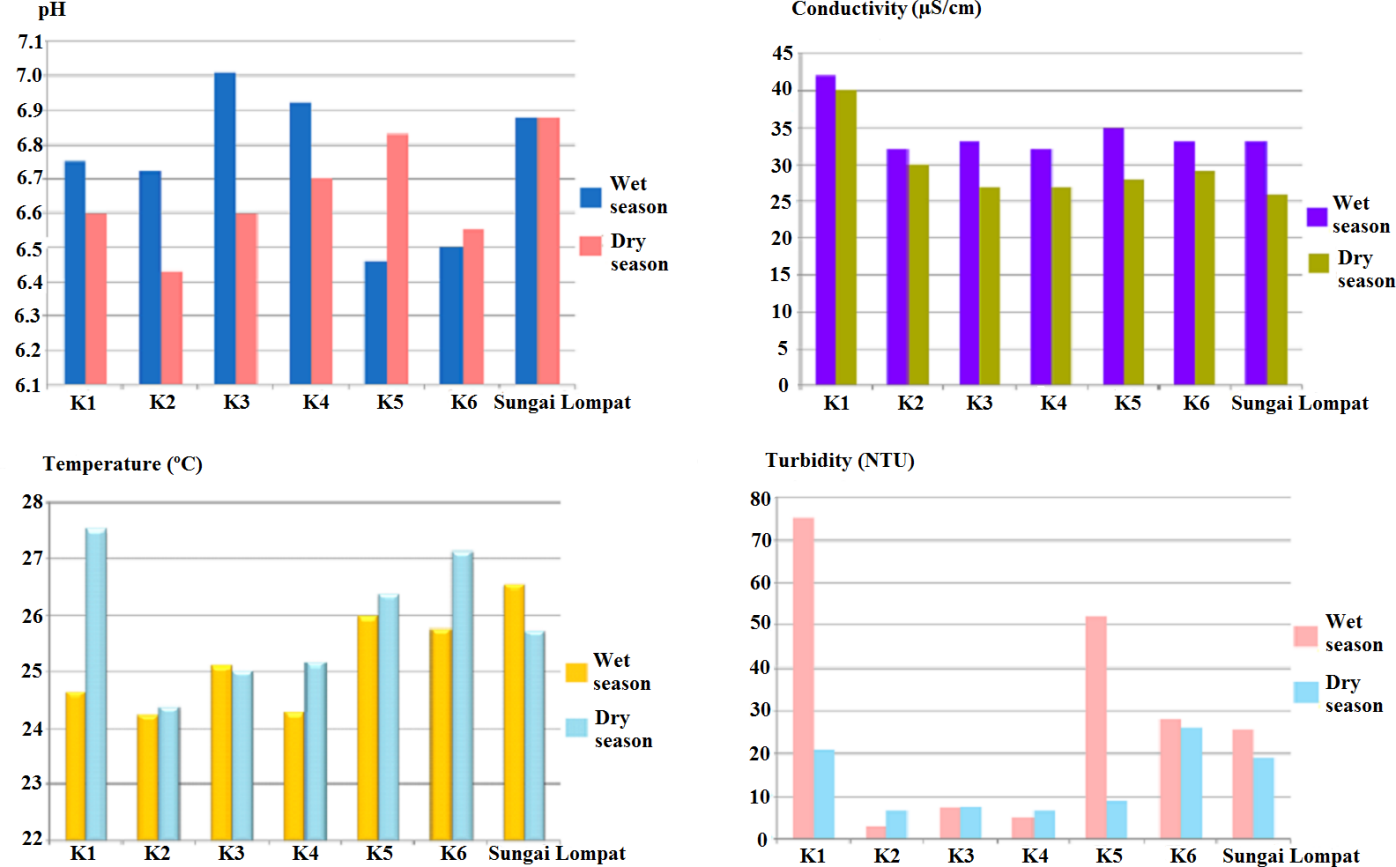
Physical parameters (pH, conductivity, temperature and turbidity) at all river sampling points.

## Results

### Physicochemical data and faecal coliforms count of the rivers

**Figure 3:**
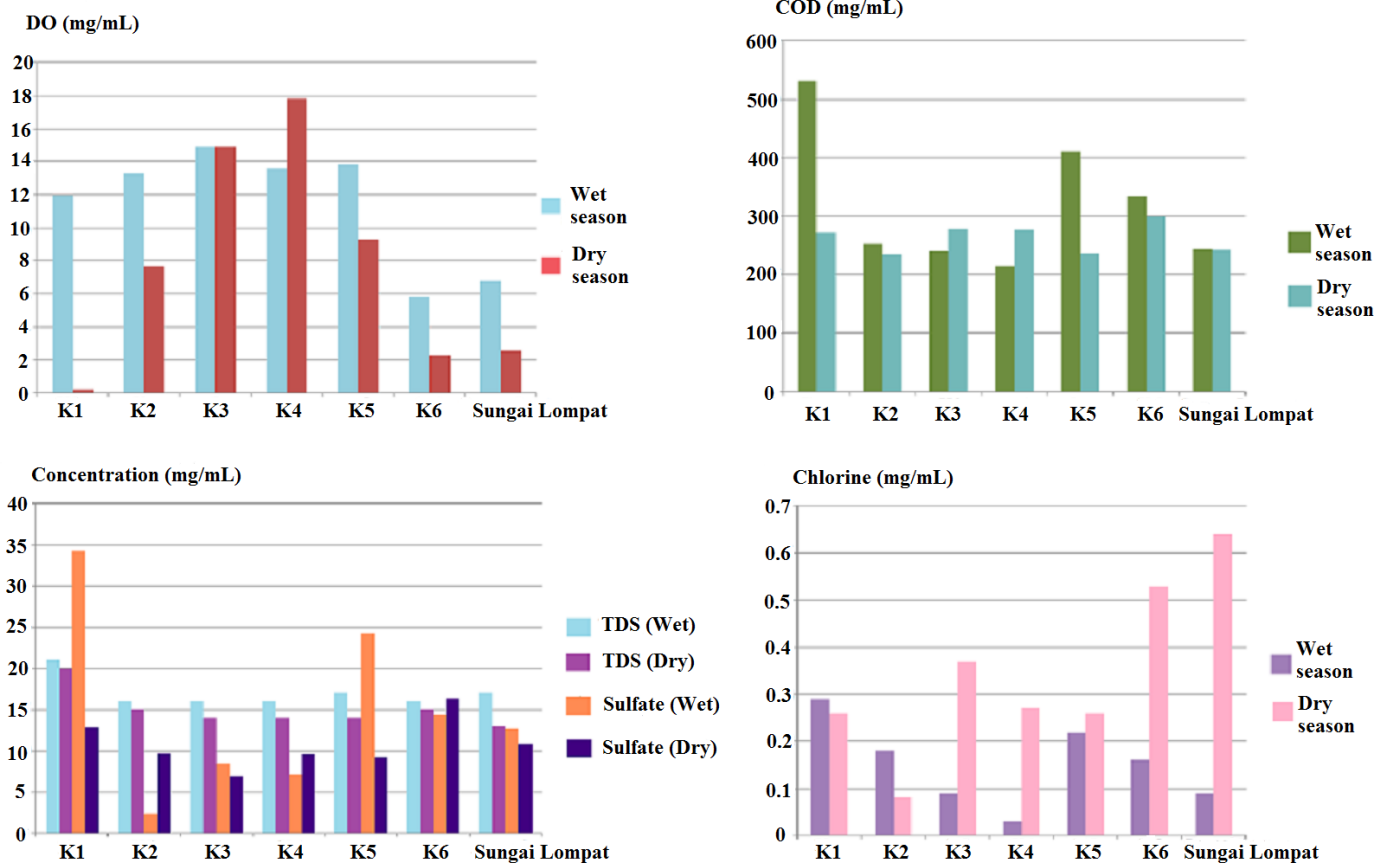
Chemical parameters (concentration of Dissolved Oxygen (DO), Chemical Oxygen Demand (COD), Total Dissolved Solids (TDS), Sulfate & Chlorine) at all river sampling points.

The physical parameters data of Sungai Krau and Sungai Lompat during both season are presented in Fig. 2 and chemical data of the rivers were as shown in Fig. 3. Physical parameters were pH, conductivity (µS/cm), temperature (°C) and turbidity (NTU). Meanwhile, chemical parameters measured were dissolved oxygen (DO) (mg/mL), chemical oxygen demand (COD) (mg/mL), total dissolved solids (TDS) (mg/mL), sulfate and total chlorine (mg/mL).

The highest reading of pH (7.01), conductivity (42.00 µS/cm) and turbidity (75.00 NTU) were recorded in K1 during the wet season. Temperature of the river water was highest in Sungai Lompat (26.55 °C). During the dry season, K1 showed the highest reading of conductivity (40.00 µS/cm) and temperature (27.53 °C). pH reading was highest in Sungai Lompat with the reading of 6.88 and the most turbid point during the dry season was K6 (26.23 NTU).

During the wet season, K1 is the point with the highest reading of chemical oxygen demand (531.89 mg/mL), total dissolved solids (21.00 mg/mL) concentration of sulfate (34.20 mg/mL) and total chlorine (0.29 mg/mL). Dissolved oxygen was highest in K3 with a reading of 14.90 mg/mL. Meanwhile, during the dry season, the highest reading in K1 was total dissolved solids (19.98 mg/mL). The reading of chemical oxygen demand (300.16 mg/mL) and sulfate (16.34 mg/mL). Dissolved oxygen was highest in K4 with reading of 17.82 mg/mL. Sungai Lompat were recorded as the sampling point of the river with the highest total chlorine reading (0.64 mg/mL) were highest in K6.

**Figure 4:**
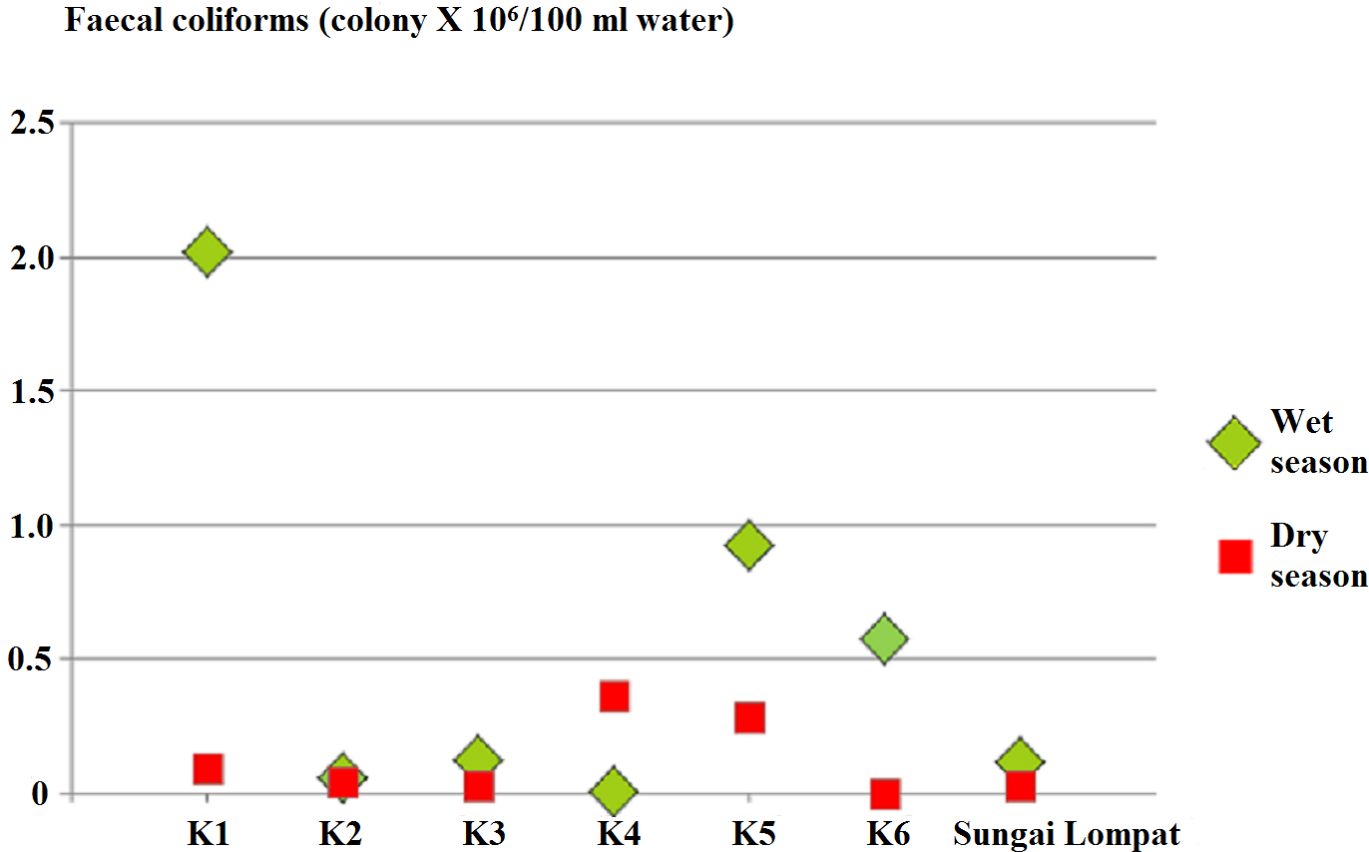
Faecal coliforms count at all river sampling points.

Figure 4 showed faecal coliforms counts in all sampling points with the highest count in K4 during the wet season (2.0180 × 10^6^) CFU per 100 mL of water . Similar to wet season faecal coliforms were positive at all sampling points in the dry season with the highest count in K4 (0.3685 × 10^6^) CFU per 100 mL.

### Detection of *Blastocystis* sp. subtypes

During the wet season, all water samples collected from the seven points of the two rivers were positive for *Blastocystis* sp. (Table 1). Only three subtypes of *Blastocystis* sp. were isolated from the water samples and they were *Blastocystis* sp. ST1, ST2 and ST3. *Blastocystis* sp. ST3 was isolated from river water samples in all seven points of the two rivers 100.0% (7/7); single occurrence of *Blastocystis* sp. ST3 was observed in K3 and K4. No single occurrence of *Blastocystis* sp. ST1 and ST2 was observed in the water samples; mixed subtypes of *Blastocystis* sp. ST2 and ST3 were detected in K1 and K5, whereby mixed subtypes of *Blastocystis* sp. ST1 and ST3 were examined in K2, K6 and Sungai Lompat.

**Table 1:** *Blastocystis* sp. subtypes isolated from river water samples during wet and dry seasons.

Point of sampling	*Blastocystis* sp. subtypes					
	ST1		ST2		ST3	
	Wet	Dry	Wet	Dry	Wet	Dry
Sungai Krau K1	−	−	+	−	+	+
Sungai Krau K2	+	−	−	−	+	+
Sungai Krau K3	−	−	−	−	+	+
Sungai Krau K4	−	−	−	−	+	+
Sungai Krau K5	−	−	+	−	+	+
Sungai Krau K6	+	−	−	−	+	+
Sungai Lompat	+	−	−	−	+	+
Total	3/7 (42.9%)	0/7 (0.0%)	2/7 (28.6%)	0/7 (0.0%)	7/7 (100.0%)	7/7 (100.0%)

**Notes.**

+ Positive for *Blastocystis* sp − Negative for *Blastocystis* sp

**Table 2:** *Blastocystis* sp. subtypes in water from other sampling sources during wet and dry seasons.

			Positive for *Blastocystis* sp. subtypes							
	Total number of samples		ST1		ST2		ST3		Other ST	
Point of sampling	Wet	Dry	Wet	Dry	Wet	Dry	Wet	Dry	Wet	Dry
Tap water	**8**	13	**1**	0	**0**	0	**3**	6	**0**	0
Water in tank (Life Saver)	**1**	1	**1**	0	**0**	0	**1**	0	**0**	0
Well	**2**	3	**1**	0	**1**	0	**2**	3	**0**	0
Stored water	**4**	2	**0**	0	**0**	0	**4**	1	**1 (ST4)**	1 (ST8)
Small stream	**0**	1	**NA**	0	**NA**	0	**NA**	0	**NA**	0
Fish pond	**1**	1	**0**	1	**0**	0	**1**	0	**0**	1 (ST10)
**Total (%)**	**16**	21	**3(18.8%)**	1(4.8%)	**1(6.3%)**	0(0.0%)	**11(68.8%)**	**10**(47.6%)	**1(6.3%)**	2(9.5%)

**Notes.**

NA Not applicable

During dry season, all seven sampling points of the rivers were positive for *Blastocystis* sp. ST3 (100.0%, 7/7). *Blastocystis* sp. ST1 and ST2 at all river sampling points were absent (Table 1).

Water samples collected from various water sources during wet season showed 68.8% (11/16) were contaminated with *Blastocystis* sp. ST3, 18.8% (3/16) were contaminated with *Blastocystis* sp. ST1 while 6.3% (1/16) was contaminated with *Blastocystis* sp. ST2 and ST4 respectively (Table 2). In Kampung Terbol, during wet season, *Blastocystis* sp. ST3 was found in 2/4 of the tap water samples and in a water tank, namely Life Saver M1 System. *Blastocystis* sp. ST1 was found in the Life Saver M1 System and in 1/4 of the water samples collected from tap water. During wet season in Kampung Lubok Wong, *Blastocystis* sp. ST3 was detected in a tap water, stored water and fish pond. In Kampung Penderas, none of the tap water samples collected from government tap water was positive for *Blastocystis* sp. subtypes. However, there was a mixed subtype of *Blastocystis* sp. ST1 and ST3 and *Blastocystis* sp. ST2 and ST3 in water samples collected from wells. *Blastocystis* sp. ST3 was found in 3/3 of water stored in closed and opened containers. *Blastocystis* sp. ST4 was detected in 1/3 of the stored water (Table 2).

During the dry season, the occurrence rate of *Blastocystis* sp. ST1 in the water samples collected from various water sources were reduced compared to wet season. In addition, none of the water samples collected in dry season was positive for *Blastocystis* sp. ST2 and ST4. The highest occurrence rate of *Blastocystis* sp. ST3 was observed (47.6%, 10/21), followed by *Blastocystis* sp. ST1 (4.8%, 1/21) and other subtypes which include *Blastocystis* sp. ST8 and ST10 (9.5%, 2/21). In tap water samples, *Blastocystis* sp. ST3 was detected in Kampung Terbol and Kampung Lubok Wong, whereby no occurrence of any *Blastocystis* sp. subtypes was observed in tap water samples of Kampung Penderas. *Blastocystis* sp. ST3 and ST8 were detected in the water stored outside a house in Kampung Penderas, and mixed subtype infections of *Blastocystis* sp. ST1 and ST10 were detected in the water collected from a small fish pond in Kampung Lubok Wong. *Blastocystis* sp. ST3 was detected in water from wells collected in Kampung Penderas and Kampung Terbol.

Tables 3A and 3B show the details of the water samples collected with the accession number and allele identity.

**Table 3:** (A) Subtyping of *Blastocystis* sp. for water samples collected during the wet season. (B) Subtyping of *Blastocystis* sp. for water samples collected during the dry season.

Samples code	Source of samples	Subtype	Identity (%)	Accession number	18S rRNA allele
(A)					
K1 B4 KT	River	2	99	KX352014	15
K2 in KT	River	1	99	KX352015	4
K2 in KT	River	3	99	KX352016	34
K3 B4 LW	River	3	99	KX352017	34
K4 in LW	River	3	99	KX352018	38
K5 B4 KP	River	2	99	KX352019	15
K5 B4 KP	River	3	99	KX352020	34
K6 in KP	River	1	99	KX352021	4
K6 in KP	River	3	99	KX352022	34
SL	River	1	99	KX352023	4
22 KP	Tap water	3	99	KX352024	38
Stored water ASPLW	Stored water	3	99	KX352025	38
Well Abu3	Well	3	99	KX352030	38
Fish pond3	Fish pond	3	99	KX352032	38
Stored water Selamat3KP	Stored water	3	99	KX352027	31
Untreated tap water hill KT	Tap water	3	99	KX352028	31
Well KP	Well water	1	99	KX352029	4
Well Abu2	Well water	2	99	KX352031	15
Stored water Selamat4	Stored water	4	99	KX352026	94
(B)					
K1 B4 KTA	River	3	99	KX351998	34
K2 in KTA	River	3	99	KX351999	34
K3 B4 LWA	River	3	99	KX352000	34
K4 in LWA	River	3	99	KX352001	34
K5 B4 KPA	River	3	99	KX352002	34
K6 in KPA	River	3	99	KX352003	34
SLa	River	3	99	KX352004	34
Well water KPA	Well water	3	99	KX352005	36
Stored water Selamat3	Stored water	3	99	KX352009	34
TBLWA	Tap water	3	99	KX352010	33
Tap water LWA	Tap water	3	99	KX352011	33
Well water KTA	Well water	3	99	KX352012	34
Fish pond LWA10	Fish pond	10	99	KX352006	43
Fish pond LWA1	Fish pond	1	99	KX352007	4
Stored water Selamat8	Stored water	8	99	KX352008	21

### Correlation of *Blastocystis* sp. subtypes with physicochemical parameters, faecal coliforms and monthly total rainfall volume

The correlation of *Blastocystis* sp. subtypes occurrence in the river with physicochemical parameters, faecal coliforms and monthly total rainfall data are shown in Table 4. During the wet season, there is a significant correlation of the occurrence of *Blastocystis* sp. ST2 and conductivity (*r*_*s*_ = 0.828, *P* < 0.05), turbidity (*r*_*s*_ = 0.791, *P* < 0.05), chemical oxygen demand (*r*_*s*_ = 0.791, *P* < 0.05), total chlorine (*r*_*s*_ = 0.798, *P* < 0.05), sulfate (*r*_*s*_ = 0.791, *P* < 0.05) and faecal coliforms (*r*_*s*_ = 0.791, *P* < 0.05). However, there is no significant correlation between *Blastocystis* sp. ST1 occurrence and the other physicochemical parameters, faecal coliform and monthly total rainfall. Correlations cannot be performed for *Blastocystis* sp. ST3 with all the parameters measured since *Blastocystis* sp. ST3 was detected at all river sampling points. During the dry season, correlation analysis cannot be performed for the occurrence of *Blastocystis* sp. ST1 and ST2 with the physicochemical parameters, faecal coliforms and monthly rainfall because of the absence of both subtypes at all river sampling points. Due to detection of *Blastocystis* sp. ST3 in all sampling points of the rivers, no correlation analysis was performed with all the parameters measured.

**Table 4:** Correlations between different *Blastocystis* sp. subtypes with physicochemical parameters, faecal coliforms and monthly total rainfall at Sungai Krau and Sungai Lompat (at 0.05 level, 2-tailed).

	Correlations					
	ST1		ST2		ST3	
Parameter	Wet	Dry	Wet	Dry	Wet	Dry
**Physical**						
pH	*r*_*s*_ = − 0.289	NA	*r*_*s*_ = − 0.474	NA	NA	NA
	*P* > 0.05		*P* > 0.05			
Conductivity	*r*_*s*_ = − 0.378	NA	*r*_*s*_ = 0.828^*^	NA	NA	NA
	*P* > 0.05		*P* < 0.05			
Temperature	*r*_*s*_ = 0.144	NA	*r*_*s*_ = 0.158	NA	NA	NA
	*P* > 0.05		*P* > 0.05			
Turbidity	*r*_*s*_ = − 0.289	NA	*r*_*s*_ = 0.791^*^	NA	NA	NA
	*P* > 0.05		*P* < 0.05			
**Chemical**						
Dissolved oxygen (DO)	*r*_*s*_ = − 0.722	NA	*r*_*s*_ = 0.158	NA	NA	NA
	*P* > 0.05		*P* > 0.05			
Chemical oxygen demand (COD)	*r*_*s*_ = 0.000	NA	*r*_*s*_ = 0.791^*^	NA	NA	NA
	*P* > 0.05		*P* < 0.05			
Total dissolved solid (TDS)	*r*_*s*_ = 0.000	NA	*r*_*s*_ = 0.683	NA	NA	NA
	*P* > 0.05		*P* > 0.05			
Total chlorine	*r*_*s*_ = − 0.073	NA	*r*_*s*_ = 0.798^*^	NA	NA	NA
	*P* > 0.05		*P* < 0.05			
Sulfate	*r*_*s*_ = − 0.289	NA	*r*_*s*_ = 0.791^*^	NA	NA	NA
	*P* > 0.05		*P* < 0.05			
Faecal coliform	*r*_*s*_ = − 0.289	NA	*r*_*s*_ = 0.791^*^	NA	NA	NA
	*P* > 0.05		*P* < 0.05			
Monthly total rainfall	*r*_*s*_ = − 0.433	NA	*r*_*s*_ = 0.000	NA	NA	NA
	*P* > 0.05		*P* > 0.05			

**Notes.**

*r*_*s*_ Correlation coefficients *P* Probability level

* significant correlation at 0.05 level.

## Discussion

Most rural and remote communities still use untreated water either from streams, rivers and wells for drinking and other daily activities (Jagals, 2006; Whelan & Willis, 2007). Many studies have implicated the role of contaminated water, especially drinking water, surface water and others as a source of *Blastocystis* sp. infections (Tan, Suresh & Smith, 2008). Cysts of *Blastocystis* sp. were detected in sewage samples collected from Kuala Lumpur, Malaysia (Suresh, Smith & Tan, 2005). A study was performed to determine the presence of *Blastocystis* sp. in water from rivers located in recreational areas in Malaysia namely Sungai Congkak and Sungai Batu. The study reported the average percentage of *Blastocystis* sp. detections of 33.3% in Sungai Congkak and 22.1% in Sungai Batu, with the highest detection rate in the downstream sampling point (Ithoi et al., 2011). *Blastocystis* sp. ST1, ST3 and ST5 were detected in rivers and lakes around Klang Valley, Malaysia (Suresh, Tan & Illi, 2009). Two previous studies in Malaysia have reported absence of a proper piped water supply and drinking unboiled or untreated water as the significant risk factors in the acquisition of *Blastocystis* sp. infections (Abdulsalam et al., 2012; Anuar et al., 2013). As one of the most common intestinal parasitic infections among the aborigines (Abdulsalam et al., 2012; Anuar et al., 2013), the occurrence of *Blastocystis* sp. in the water sources should be implicated as the source of *Blastocystis* sp. infections in humans and animals. In addition, certain *Blastocystis* sp. subtypes including *Blastocystis* sp. ST1 (Moosavi et al., 2012), ST2 (Ramírez et al., 2014), ST3 (Tan, Suresh & Smith, 2008) and ST4 (Dominguez-Marquez et al., 2009) have been reported to be pathogenic. Therefore, there is a need to detect the occurrence of the *Blastocytis* sp. subtypes in the water samples used by the aborigines in this study.

This study reveals that river water used by the villagers are highly contaminated with human and animal faecal materials as shown by the detection of high faecal coliform counts in all seven sampling points in Sungai Krau and Sungai Lompat. Interestingly, *Blastocystis* sp. ST1, ST2 and ST3 were the subtypes identified in the river water samples of which *Blastocystis* sp. ST3 being the most predominant subtype isolated in all water sampling points. Besides that, *Blastocystis* sp. ST3 was the only subtype that was persistently isolated during wet and dry seasons. In contrast, a study in Nepal highlighted four of the river water samples were positive for *Blastocystis* sp. ST1 and ST4 (Lee et al., 2012). Abdulsalam (2013) in her thesis reported *Blastocystis* sp. ST4 was the only subtype isolated from river water samples. Of the nine *Blastocystis* sp. subtypes reported in humans, four subtypes (ST1–ST4) are common in humans (Clark et al., 2013). However, *Blastocystis* sp. ST4 was rarely reported outside Europe (Alfellani et al., 2013). This study detected ST4 in one of the samples collected from water stored in a container during the wet season. Based on the lack of reports of ST4 in Malaysia, the potential of faeces of the aborigines to contaminate the water sample in the container was low. The presence of *Blastocystis* sp. ST1, ST2 and ST3 isolated from river water samples during wet season and *Blastocystis* sp. ST3 during dry season in all of the sampling points in this study, in addition to the detection of faecal coliforms in all river water samples indicates that the sources of river water contamination by *Blastocystis* sp. are most possibly from humans and a small amount from animal faeces. These findings were supported by many molecular studies in humans that revealed the common *Blastocystis* sp. subtypes were ST1–ST4 with the most dominant subtype being ST3 (Yoshikawa et al., 2004; Özyurt et al., 2008; Tan, Ong & Suresh, 2009; Yakoob et al., 2010; Nithyamathi, Chandramathi & Kumar, 2016).

Although the community in Kampung Penderas is equipped with proper tap water supply and toilet facilities by the government, data gathered from the questionnaires and from our own observations revealed that many of the villagers still practise open defaecation and collect water from the rivers and wells for daily activities. Water is kept in a container for daily usage and sometimes kept for further usage during a shortage of water. The less-structured villages of Kampung Terbol and Kampung Lubok Wong are not equipped with safe pipe water supply and toilets. The aboriginal community in these two villages built up their own piping system directly from streams at the hilly areas since the water from the upstream is cleaner and less polluted than the Sungai Krau itself. Some of the aborigines still collect water from Sungai Krau for storage and to be used when there is a shortage of water from their own piping system. Besides own-built piping system, Kampung Terbol and Kampung Lubok Wong are equipped with Life Saver M1 System. The tank operated without chemicals or power supply and filtered any water sources including rain water, well water and water from the nearby river. Realizing the chances of contamination of other water sources with *Blastocystis* sp., other water sources used by the community including water in wells, tank, fish pond, stored containers and tap waters were also sampled. In this study, most of the water collected from many sources during wet season was contaminated by *Blastocystis* sp. ST1, ST2 and ST3, with *Blastocystis* sp. ST3 as the most prevalent subtype in the water within the vicinity of the aboriginal dwellings.

*Blastocystis* sp. ST3 has been nailed down as the pathogenic subtype of *Blastocystis* sp. (Jones Ii et al., 2008). Therefore, the detection of this particular subtype persistently in the river water samples and other water sources including tap water, wells and water stored in container during both seasons may be an important point to be raised. Further investigations need to be performed to determine *Blastocystis* sp. infections in the aborigines, so that the possibility of waterborne transmission of the subtype can be ruled out.

During dry season, there was a marked reduction in the detection rate of *Blastocystis* sp. ST1 and absence of *Blastocystis* sp. ST2 and ST4 in the water samples collected from various water sources in the villages. However, there were additional *Blastocystis* sp. subtypes of ST8 and ST10 detected in the water samples collected from stored water and fish pond. *Blastocystis* sp. ST8 was rarely reported in humans, whereby *Blastocystis* sp. ST10 was identified in primates and artiodactyls (Stensvold et al., 2009; Ramírez et al., 2014). Therefore, we postulate that both *Blastocystis* sp. subtypes were from animal source which might contaminate the water. The *Blastocystis*-positive stored water which was used for washing hands and legs was left outside the house in an uncovered container. Therefore, the chance of any animals to contaminate the water cannot be avoided which might explain the discovery of *Blastocystis* sp. ST8 in one of the stored water collected. The occurrence of *Blastocystis* sp. ST10 in the water collected from fish pond was postulated to be from the faeces of fishes. However, since *Blastocystis* sp. ST10 is found mostly in primates and cattle, this could suggest accidental colonisation of the subtype in fishes or contamination of the water used to fill the fish pond with *Blastocystis* sp. ST10 by the faeces of primates or cattles.

Our findings revealed that there were no *Blastocystis* sp. subtypes detected in water samples collected from treated governmental tap water in Kampung Penderas although most of the tap water supplies in Malaysia originated from river waters. Sedimentation and chlorination in the water treatment process was found to be able to remove and kill *Blastocystis* sp. cyst although a study done by Zaki, Zaman & Khan (1996) reported that this protist showed resistance to chlorination. In contrast, *Blastocystis* sp. ST1 and ST3 were detected in untreated tap water from Kampung Terbol during wet season and single occurrence of *Blastocystis* sp. ST3 in untreated tap water from Kampung Lubok Wong. During dry season, *Blastocystis* sp. ST3 was detected in untreated tap water in Kampung Terbol and Kampung Lubok Wong. Therefore, the untreated tap water could be one of the sources of *Blastocystis* sp. infection in these two villages. Since many of the rural communities may still use untreated water from streams, rivers and wells, so this study may highlight the importance of drinking treated and boiled water.

To the best of our knowledge, this is the first study done in Malaysia to provide data on the seasonal influence on the presence of *Blastocystis* sp. in water sources. The worst floods in Malaysia which were affected by the new moon phenomenon and perigee hit the north-eastern parts of the country starting from mid of December 2014 to early January 2015 (Abdullah et al., 2014 ; Wei, 2014; Baharuddin et al., 2015; Ambu, 2015) might have changed the distribution of *Blastocystis* sp. subtypes in the study area. During wet season, *Blastocystis* sp. isolated in six sampling points of Sungai Krau and a point at Sungai Lompat, as well as in water samples collected from various water sources in the villages were of *Blastocystis* sp. ST1–ST3. However, to our surprise, there was a predominance of *Blastocystis* sp. ST3 at all river sampling points and in water samples collected from various other water sources in the villages during dry season. In addition, there was an absence of *Blastocystis* sp. ST1 and ST2 in the river water samples. In other water sources, there was a reduction in *Blastocystis* sp. ST1 occurrence and absence of *Blastocystis* sp. ST2. These findings lead to our postulation that *Blastocystis* sp. ST1 and ST2 might not be able to withstand the adverse condition and were flushed during the heavy flood. The detection of *Blastocystis* sp. ST3 as the most prevalent subtype especially during dry season, which was five months after the heavy flood suggest that it is the most resistant subtype since it is able to survive harsh environmental conditions especially during the heavy flood.

The possibility of waterborne transmission of *Blastocystis* sp. to humans in this study is not only restricted to the aboriginal community, however, since Sungai Krau flows along the Malay community which is located before Kampung Terbol (at the more upstream area) and after Kampung Penderas (at the more downstream area) as well, so the chances of waterborne transmission to the communities living along the rivers were possible. In addition, during water sampling in both seasons, we observed few groups of people from various other places went to the Sungai Krau and Sungai Lompat for fishing, since most of them search for large freshwater fishes. Therefore, the possibility of infection from the fishes which might as well become infected with *Blastocystis* sp. is high in those groups of people, especially when the fishes are not properly cleaned and cooked. However, since we did not take samples from the fishes, we do not know whether the fishes pose a risk as a source of *Blastocystis* sp. infections to these groups of people.

The primer BhRDr and RD5 were chosen in this study since they can identify *Blastocystis* sp. both at ST levels and 18S allele analysis with no chance of any subtype being missed in the detection (Stensvold, 2013). Different subtypes display various level of intra-subtype diversity where *Blastocystis* sp. ST3 is known to exhibit the most substantial intra-subtype genetic diversity (Stensvold, Alfellani & Clark, 2012; Stensvold, 2013). This study indicated that there were three ST3 allele found in the water samples collected during the wet season (ST3 allele 31, 34 and 38), meanwhile in the dry season, there were also three allele identified; ST3 allele 33, 34 and 36. *Blastocystis* sp. ST3 allele 34 was reported as a quite common allele in humans (Alfellani et al., 2013; Pandey et al., 2015; Das et al., 2016), therefore the detection of the allele in the water samples during both seasons in this study might indicate the contamination of water sources with human faecal samples. *Blastocystis* sp. ST3 allele 34, 36 and 38 have been reported in human and non-human primates (Alfellani et al., 2013), therefore both human and animal faecal samples might contribute to the contamination of the water samples with *Blastocystis* sp. during the wet and dry seasons.

Correlations between *Blastocystis* sp. subtypes occurrence in the rivers and physicochemical parameters and faecal coliforms showed significant positive correlation between the presence of *Blastocystis* sp. ST2 in the river water and conductivity, turbidity, chemical oxygen demand, total chlorine, sulfate and faecal coliforms (*p* < 0.05). This shows that this particular subtype is able to survive at more polluted water. However, *Blastocystis* sp. ST1 and ST3 are able to survive in any condition of the water, whether less or more polluted water. Both *Blastocystis* sp. ST1 and ST3 are not affected by physicochemical changes of the water. Among the three subtypes detected in the river water samples, only *Blastocystis* sp. ST3 is able to sustain and grow in the water after many months regardless of the environmental conditions.

## Conclusion

The river water samples used by the aboriginal community were highly contaminated with organic materials and faeces of humans and animals. The occurrence of *Blastocystis* sp. in the water in this study hopes to raise awareness on the importance of the consumption of treated or boiled water and the need to improve sewage disposal system in the community. Seasonal variation plays a role in the occurrence and survival of *Blastocystis* sp. subtypes in the water samples. Among all the subtypes present in the water samples, *Blastocystis* sp. ST3 is suggested to be the most resistant and robust subtype and able to survive harsh environmental conditions since it was found in the river and other water sources in both seasons. The presence of *Blastocystis* sp. ST2 in the river water samples were significantly correlated with certain physicochemical parameters during wet season and this brings to the suggestion that *Blastocystis* sp. ST2 survives in more polluted and contaminated water. *Blastocystis* sp. ST1 and ST3 occurrence are not affected by any physicochemical changes of the water. Since faecal coliforms were detected at all sampling points of the rivers, therefore the possible sources of *Blastocystis* sp. contaminations could therefore be from the faeces of humans or animals. Avoidance of *Blastocystis* sp. infection and spread to wider areas especially by waterborne route to other communities require a collaborative effort from different authorities and communities. Health education, treated and safe water supply and toilets were among the strategies to be applied in order to obliterate the chances of *Blastocystis* sp. transmissions to humans and animals from water sources.

##  Supplemental Information

10.7717/peerj.2541/supp-1 Supplemental Information 1 Details of the *Blastocystis*-positive water samples collected during the wet (table A) and dry (table B) seasons in aboriginal settlement

## Additional Information and Declarations

### Field Study Permissions

The following information was supplied relating to field study approvals (i.e., approving body and any reference numbers): Permission for water collection from the aboriginal dwellings were obtained from the Ministry of Rural and Regional Development Malaysia (reference number: JAKOA/PP.30.032Jld29(04).

### DNA Deposition

The following information was supplied regarding the deposition of DNA sequences: The sequence obtained were deposited in GenBank (accession numbers : KX351998–KX352032).

### Data Availability

The following information was supplied regarding data availability: The raw data has been supplied as a Supplemental Information 1.
