# Supplementary material for: Occurrence of Blastocystis sp. in water catchments at Malay villages and Aboriginal settlement during wet and dry seasons in Peninsular Malaysia
Source: PeerJ. 2016 Oct 6;4:e2541. doi: 10.7717/peerj.2541 (PMC5068341; doi:10.7717/peerj.2541)
Supplement: Supplemental Information 1 [file peerj-04-2541-s001.docx]

**Supplementary tables**

Table A Result from BLAST and pubmlst for samples collected during the wet season

| Samples code | Source of samples | Query cover (%) | Identity (%) | Most similar sequence for GenBank | Subtype | Accession number | 18S rRNA allele |
| --- | --- | --- | --- | --- | --- | --- | --- |
| K1 B4 KT | River | 98 | 99 | KM374619.1 | 2 | KX352014 | 15 |
| K2 in KT | River | 99 | 99 | KF848553.1 | 1 | KX352015 | 4 |
| K2 in KT | River | 99 | 99 | KR000003.1 | 3 | KX352016 | 34 |
| K3 B4 LW | River | 99 | 99 | KR000003.1 | 3 | KX352017 | 34 |
| K4 in LW | River | 99 | 99 | HQ909889.1 | 3 | KX352018 | 38 |
| K5 B4 KP | River | 99 | 99 | KM374619.1 | 2 | KX352019 | 15 |
| K5 B4 KP | River | 99 | 99 | KR000003.1 | 3 | KX352020 | 34 |
| K6 in KP | River | 99 | 99 | KF848553.1 | 1 | KX352021 | 4 |
| K6 in KP | River | 99 | 99 | KR000003.1 | 3 | KX352022 | 34 |
| SL | River | 99 | 99 | KF848553.1 | 1 | KX352023 | 4 |
| 22 KP | Tap water | 100 | 99 | HQ909889.1 | 3 | KX352024 | 38 |
| Stored water ASPLW | Stored water | 100 | 99 | HQ909889.1 | 3 | KX352025 | 38 |
| Well Abu3 | Well | 99 | 99 | HQ909889.1 | 3 | KX352030 | 38 |
| Fish pond3 | Fish pond | 99 | 99 | HQ909889.1 | 3 | KX352032 | 38 |
|  |  |  |  |  |  |  |  |
| Stored water Selamat3KP | Stored water | 99 | 99 | HQ909889.1 | 3 | KX352027 | 31 |
| Untreated tap water hill KT | Tap water | 99 | 99 | HQ909889.1 | 3 | KX352028 | 31 |
| Well KP | Well water | 99 | 99 | KF848553.1 | 1 | KX352029 | 4 |
|  |  |  |  |  |  |  |  |
| Well Abu2 | Well water | 98 | 99 | KM374619.1 | 2 | KX352031 | 15 |
| Stored water Selamat4 | Stored water | 99 | 99 | JN682513.1 | 4 | KX352026 | 94 |
|  |  |  |  |  |  |  |  |

Table B Result from BLAST and pubmlst for samples collected during the dry season

| Samples code | Source of samples | Query cover (%) | Identity (%) | Most similar sequence for GenBank | Subtype | Accession number | 18S rRNA allele |
| --- | --- | --- | --- | --- | --- | --- | --- |
| K1 B4 KTA | River | 96 | 99 | KR000003.1 | 3 | KX351998 | 34 |
| K2 in KTA | River | 98 | 99 | [KP284171.1](http://www.ncbi.nlm.nih.gov/nucleotide/928008212?report=genbank&log$=nucltop&blast_rank=18&RID=SY2YNU9C013) | 3 | KX351999 | 34 |
| K3 B4 LWA | River | 99 | 99 | KR000003.1 | 3 | KX352000 | 34 |
| K4 in LWA | River | 98 | 99 | KR000003.1 | 3 | KX352001 | 34 |
| K5 B4 KPA | River | 100 | 99 | KR000003.1 | 3 | KX352002 | 34 |
| K6 in KPA | River | 100 | 99 | KR000003.1 | 3 | KX352003 | 34 |
| SLa | River | 96 | 99 | KR000003.1 | 3 | KX352004 | 34 |
| Well water KPA | Well water | 97 | 99 | KR000003.1 | 3 | KX352005 | 36 |
| Stored water Selamat3 | Stored water | 97 | 99 | KR000003.1 | 3 | KX352005 | 34 |
| TBLWA | Tap water | 99 | 99 | HQ909891.1 | 3 | KX352010 | 33 |
| Tap water LWA | Tap water | 99 | 99 | HQ909891.1 | 3 | KX352011 | 33 |
| Well water KTA | Well water | 98 | 99 | KP284171.1 | 3 | KX352012 | 34 |
| Fish pond LWA10 | Fish pond | 99 | 99 | KC148207.1 | 10 | KX352006 | 43 |
| Fish pond LWA1 | Fish pond | 98 | 99 | KF848570.1 | 1 | KX352007 | 4 |
| Stored water Selamat8 | Stored water | 99 | 99 | JQ974945.1 | 8 | KX352008 | 21 |
